# Efficacy and safety of underwater endoscopic mucosal resection for ≤20 mm superficial non-ampullary duodenal epithelial tumors: Systematic review and meta-analysis

**DOI:** 10.3389/fmed.2022.1077806

**Published:** 2023-01-06

**Authors:** Jixiang Liu, Shaojie Duan, Yichong Wang, Hongye Peng, Youjia Kong, Shukun Yao

**Affiliations:** ^1^Graduate School of Beijing University of Chinese Medicine, Beijing, China; ^2^Department of Gastroenterology, China-Japan Friendship Hospital, Beijing, China; ^3^Beijing Hospital of Traditional Chinese Medicine Affiliated to Capital Medical University, Beijing, China

**Keywords:** duodenum, non-ampullary adenoma, underwater endoscopic mucosal resection, efficacy, safety

## Abstract

**Background and aims:**

Superficial non-ampullary duodenal epithelial tumors (SNADETs) as a rare disease have gradually increased in recent years. Underwater endoscopic mucosal resection (UEMR) has emerged as a newly available option for the endoscopic resection of SNADETs. This study aimed to evaluate the efficacy and safety of UEMR for ≤20 mm SNADETs.

**Methods:**

A literature search was performed across multiple databases, including PubMed, Embase, Scopus, and Clinical trials for studies containing tumors ≤20 mm published from January 1, 2012, to August 8, 2022. Outcomes examined were the pooled rates of en bloc resection, R0 resection, adverse events, and recurrence. Subgroup analyses of the resection rate were conducted stratified by sample size and polyp size.

**Results:**

A total of 10 studies with UEMR performed in a total of 648 tumors were included for analysis. The pooled rate of en bloc resection and R0 resection was 88.2% (95% confidence interval (CI): 82.1–93.2) and 69.1% (95% CI: 62.2–76.1), respectively. The results showed pooled rate of intraoperative bleeding rate was 2.9% (95% CI: 0–9.0), delayed bleeding rate was 0.9% (95% CI: 0.1–2), recurrence rate was 1.5% (95% CI: 0–4.9). In the subgroup analysis, R0 and en-bloc resection rates were significantly higher in <10 mm than 10–20 mm SNADETs subgroups (R0 resection rate 83.1 vs. 48.6%; en bloc resection rate 100.0 vs. 84.0%, *P* < 0.05).

**Conclusion:**

Underwater endoscopic mucosal resection was an effective and safe technique for the optional treatment for ≤20 mm SNADETs, especially of <10 mm.

**Systematic review registration:**

https://www.crd.york.ac.uk/PROSPERO/, identifier CRD42022340578.

## Introduction

Superficial non-ampullary duodenal epithelial tumors (SNADETs) was rare and the incidence rate in autopsied cases was 0.02–0.5% (1–3). The number of reported primary non-ampullary duodenal carcinomas has increased gradually in recent years due to the development of endoscopic equipment and endoscopic techniques, and increased awareness of patients (4). Elimination of adenoma-carcinoma sequence at the early stage is essential, which is considered as one of the pathways in the carcinogenesis of duodenal carcinomas. Conventional endoscopic mucosal resection (CEMR) is widely applied for ≤20 mm SNADETs, which is an approach that required injecting a solution into the submucosa of target polyps to establish a safety cushion and removal with snare cautery. Nevertheless, because of a bending lumen and extremely thin muscle layer in the duodenum, it is associated with a higher rate of recurrence (0–37%), delayed bleeding (0–33%) and delayed perforation (3%) than gastric and colonic treatment (5, 6). Therefore, the effectiveness and safety of excising SNADETs with CEMR are limited.

Underwater EMR (UEMR) is a novel technique that was first introduced by Binmoeller et al. (7) in 2012. Instead of injecting submucosally before polypectomy in CEMR, the process involves filling the luminal space with water, which may lower the risk of intestinal perforation and disruption of deeper intestinal layers. However, the efficacy and safety of UEMR in the resection of SNADETs remain ambiguous. There were large variations across clinical trials in the en-bloc resection rate and adverse events ranging from 41 to 78% and ranging from 0 to 4%, respectively (8, 9), and the lack of stratification of the size of SNADETs in their performance attract criticism. Kato et al. (10) found that the en-bloc resection rate of UEMR for 1–9 mm SNADETs ranged from 89.1 to 93.3%, however, for 15–19 mm SNADETs was only 62.0%. Therefore, this study aimed to investigate the effectiveness and safety of UEMR for resection of ≤20 mm SNADETs.

## Materials and methods

### Search strategy

A comprehensive literature search was conducted in several databases, including PubMed, Embase, Scopus, and Clinical trials for studies that were published from January 1, 2012, to August 8, 2022. The search strategy was based on keywords, supplemented with standardized index terms. Keywords contained “endoscopic mucosal resection,” “Underwater EMR,” “UEMR” and phrases associated with the disease location such as “Duodenum.” The studies were searched separately by two individuals (Jixiang and Shaojie), and 1,395 citations were finally exported to the endnote. The full search strategy is available in Appendix 1. The MOOSE checklist (Preferred Reporting Items for Systematic Reviews and Meta-Analyses) was followed and is provided in Appendix 2 (11).

### Study selection

In this meta-analysis, only studies exploring the effectiveness or safety of the UEMR technique to remove SNADETs ≤20 mm were considered eligible. Studies were included regardless of sample size, study setting, and whether published as full manuscripts or abstracts, as long as the data needed for the analysis were available. Our exclusion criteria were studies that were not published in the English language. If several reports have overlap data, only the most recent or most appropriate articles were retained.

### Data extraction and study quality assessment

Articles were independently screened by two people (Jixiang and Shaojie) and the data were extracted into a standardized form. Conflicts in filtering were resolved by consensus or by a neutral person (Yichong).

The quality of the cohort study was judged by the modified Newcastle-Ottawa scale (NOS), which ranges from 0 to 6 (12). We defined the good quality of a study as a score >4 on the NOS, and 2–4 as moderate, ≤2 as poor quality. Two authors (Jixiang and Jing) evaluated these trials, respectively and recorded the score in the corresponding standardized forms.

### Definitions and outcomes evaluated

Pooled rate of en-bloc resection.

Pooled rate of R0 resection.

Pooled rate of adverse events.

Pooled rate of recurrence.

En-bloc resection, as opposed to resection in numerous parts, was defined as resection in a single piece. A specimen having a negative margin on microscopy was referred to as an R0 resection. Adverse events included intraoperative bleeding, perforation, and delayed bleeding perforation. Within 30 days of UEMR, the occurrence of bloody stool, hematemesis, or melena was presented as evidence of delayed bleeding. Delayed perforation was defined as abdominal computed tomography findings of air or luminal contents beyond the gastrointestinal system. Recurrence was defined as finding lesions within the 12 months follow-up period following the original resection.

### Statistics analysis

The meta-analysis was carried out by measuring the pooled estimates and 95% confidence intervals (CI) following the methods suggested by DerSimonian and Laird (13) using a random-effects model. Heterogeneity was assessed by using the Cochran Q statistical test and the I^2^ statistics (14–16). Values of ≤30, 30–60, 61–75, and >75% were regarded as low, moderate, substantial and considerable heterogeneity, respectively (17). Publication bias was ascertained quantitatively by the Egger test (18).

In addition, subgroup analysis of the main outcomes was conducted according to factors that may have influenced the outcomes, such as the sample size and polyp size. According to the statistical program. All analyses were performed using STATA version 15.0 (StataCorp, College Station, TX, USA). If the outcomes which we are concerned about have extreme data, such as 0 or 100%, “metaprop” software commands would be used (19), otherwise used “metan,” “metaninf,” and “metanbias.”

## Results

### Search results

**FIGURE 1:**
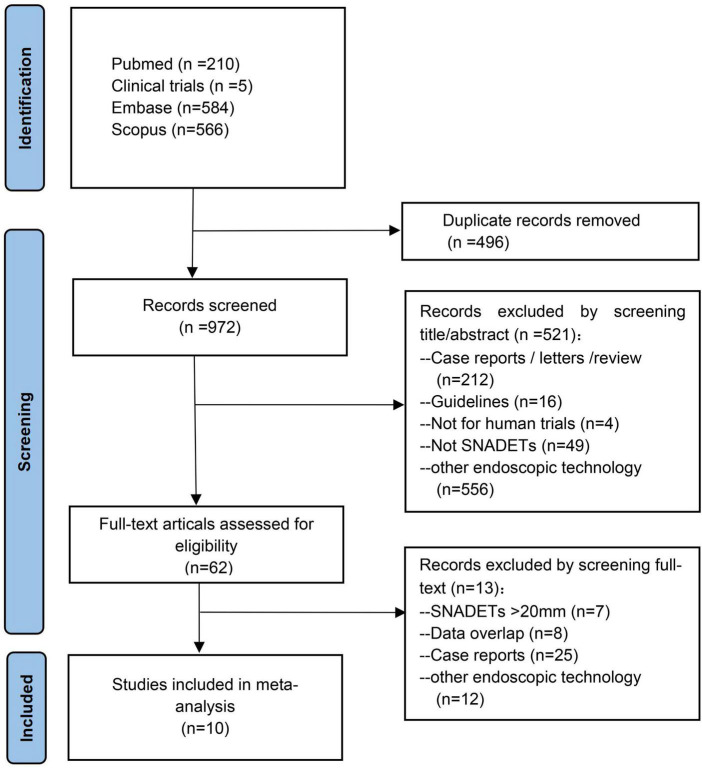
The flowchart of the selection process.

From an initial 1,395 studies, 496 studies were removed as they were duplicates and another 837 studies after screening title and abstract. Ten studies with 648 tumors were included in the final meta-analysis (20–29). A total of 648 polyps that were ≤20 mm in size were resected by UEMR. Except for two studies not specifying (23, 26), the polyps’ histopathological in the rest of the studies were adenoma with or without heterogeneous hyperplasia and carcinoma. A schematic diagram of the study selection is provided in Figure 1.

### Characteristics and quality of the studies

All of the included studies were cohort studies, of which nine were retrospective and one was prospective in design. One study was published as abstracts (26), but outcomes data were displayed. All of the studies were conducted in Japan, and two were from multicenter (28, 29). Six studies had sample sizes >50, and four studies make subgroup analyses according to polyps size. Further details and the study characteristics are described in Table 1. Overall, three studies were deemed to be of high quality, and the others were of moderate quality. Table 2 presents the detailed scores of the quality assessment.

**TABLE 1:** Original data of the included studies.

References	Country	Polys, *n*	En bloc resection	R0 resection	Delayed bleeding	Delayed perforation	Intraoperative bleeding	Intraprocedural perforation	Recurrence	Follow-up time
Hirasawa et al. (21)	Japan	66	NR	51	3	0	NR	0	NR	NR
Toya et al. (27)	Japan	17	17	15	0	0	NR	0	NR	1–65 months
Okimoto et al. (9)	Japan	61	46	31	1	0	NR	0	1	ł12 months
Furukawa et al. (20)	Japan	28	27	20	0	0	NR	0	0	2 months
Iwagami et al. (22)	Japan	134	106	NR	2	1	1	0	NR	NR
Kiguchi et al. (23)	Japan	90	78	60	2	0	NR	0	NR	NR
Shibukawa et al. (25)	Japan	16	14	NR	1	0	3	0	2	3 months
Takahashi and Oyama, (27)	Japan	56	NR	40	0	0	NR	0	0	NR
Yamasaki et al. (28)	Japan	166	149	111	2	0	4	0	4	12 months
Yamashina et al. (29)	Japan	14	13	8	0	0	1	0	NR	NR

**TABLE 2:** Study quality assessment.

References	Selection				Outcome		Score	Quality
	**Representativeness of the average adult in the community**	**Cohort size**	**Assignment of exposure**	**Outcome not present at the start**	**Assignment of outcome**	**Adequacy of follow up**	**MAX = 6**	**HIGH > 4, MEDIUM 3–4, LOW < 3**
	**Population-based: 1; multi-center: 0.5; single-center: 0**	**>50 patients: 1; 50–20: 0.5; <20: 0**	**Strictly defined records:1; not record: 0**	**not present: 1; present: 0**	**According to reliable records: 1; not: 0**	**yes: 1; not mentioned: 0**		
Furukawa et al. (20)	0	0.5	1	1	1	1	4.5	HIGH
Hirasawa et al. (21)	0	1	1	1	1	0	4	MEDIUM
Iwagami et al. (22)	0	1	1	1	1	0	4	MEDIUM
Kiguchi et al. (23)	0	1	1	1	1	0	4	MEDIUM
Okimoto et al. (24)	0	1	1	1	1	1	5	HIGH
Shibukawa et al. (25)	0	0	1	1	1	1	4	MEDIUM
Takahashi and Oyama (26)	0	1	1	1	1	0	4	MEDIUM
Toya et al. (27)	0	0	1	1	1	1	4	MEDIUM
Yamasaki et al. (28)	1	1	1	1	1	1	6	HIGH
Yamashina et al. (29)	1	0	1	1	1	0	4	MEDIUM

### Meta-analysis outcomes

#### En-bloc and R0 resection rate

**FIGURE 2:**
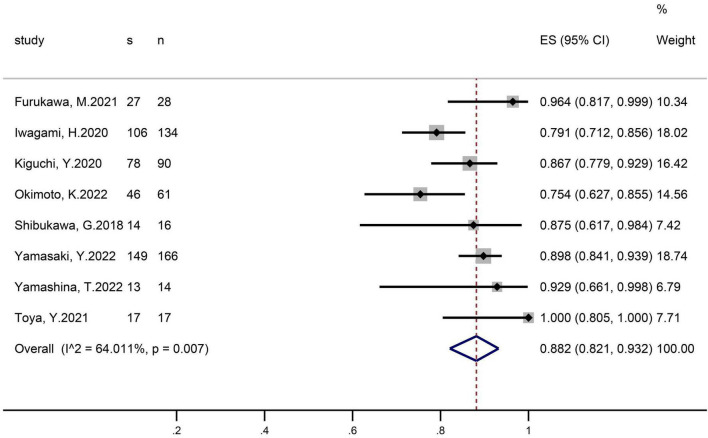
Forest plot, en-bloc resection rate.

En-bloc resection was reported in eight studies that included 526 polyps (Figure 2). The pooled proportion of patients with successful en-bloc resection was 88.2% (95% CI 82.1–93.2). The I^2^ heterogeneity was 64%.

**FIGURE 3:**
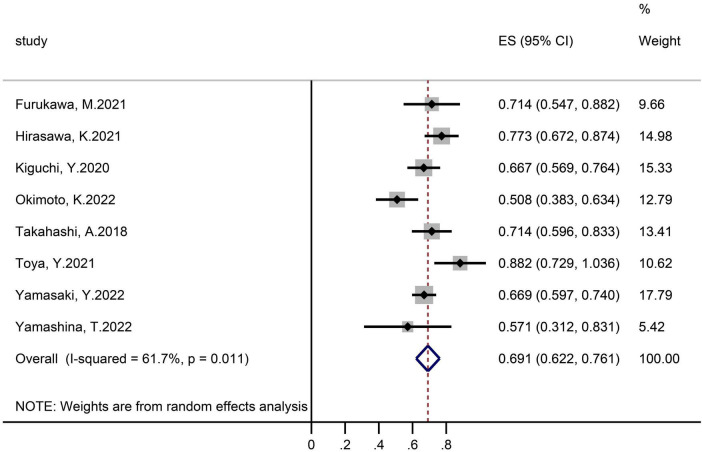
Forest plot, R0 resection rate.

Eight studies assessing 498 polyps provided eligible data on the R0 resection rate (Figure 3). The achieved proportion was 69.1% (95% CI 62.2–76.1) with an I^2^ heterogeneity of 61%.

### Adverse events

Intraoperative bleeding was recorded in four studies that evaluated 330 polyps (Supplementary Figure 1), of which pooled proportion was 2.9% (95% CI 0–9.0). The I^2^ heterogeneity was 68%. eight studies investigated the delayed-bleeding events (Supplementary Figure 2), and the pooled proportion was 0.9% (95% CI 0.1–2) with 0% I^2^ heterogeneity.

In 8 studies with 526 polyps, there were no intraoperative perforation incidents, and only one patient experienced a delayed perforation.

#### Recurrence

Data on the recurrence rate were eligible from four studies that examined 299 polyps (Supplementary Figure 3). The achieved proportion was 1.5% (95% CI 0–4.9) with an I^2^ heterogeneity of 46%.

### Subgroup analysis

#### According to the sample size

Four studies with a sample size of less than 50 including 75 polyps reported en-bloc resection rate, and the pooled proportion was 95.9% (95% CI 89.2–99.8, I^2^ = 0). Four trials with 451 lesions with a sample size of more than 50 reported the pooled en-bloc resection rate was 83.6% (95% CI 76.5–89.6, I^2^ = 71%) (Supplementary Figure 4).

In subgroups with a sample size of less than 50, three studies with 59 polyps yielded a pooled R0 resection rate which was 74.5% (95% CI 57.9–91.2, I^2^ = 58%). In subgroups with a sample size of more than 50, R0 resection rates were reported in five studies containing 439 polyps. The pooled R0 resection rate was 67.0% (95% CI 59.7–74.4, I^2^ = 63%) (Supplementary Figure 5).

#### According to polyps size

**FIGURE 4:**
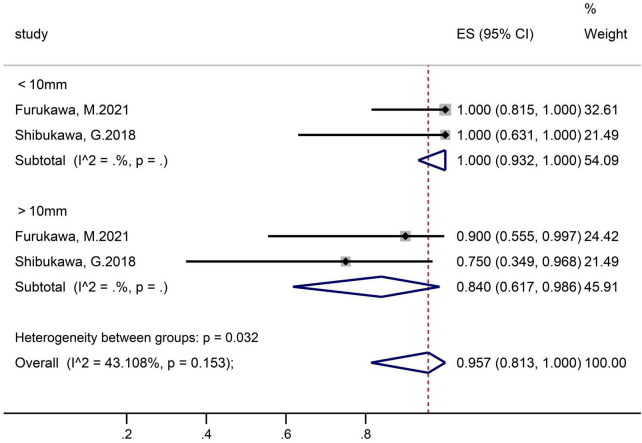
Forest plot, en-bloc resection rate, subgroup analysis according to tumor size.

Two studies that contained 26 polyps in the ≤10 mm subgroup and 15 polyps in the >10 mm subgroup reported en-bloc resection rates (Figure 4). The pooled proportion in group of ≤10 mm and >10 mm was 100% (95% CI 93.2–100) and 84.0% (61.7–98.6), respectively.

**FIGURE 5:**
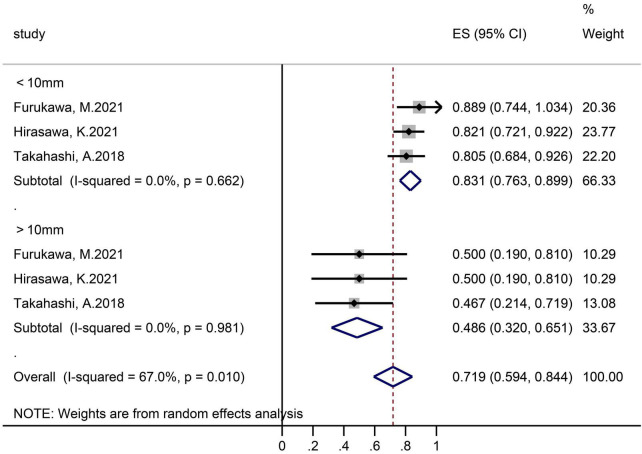
Forest plot, R0 resection rate, subgroup analysis according to tumor size.

Three studies grouped according to polyp size reported R0 resection rates (Figure 5). In the subgroups of ≤10 and >10 mm, the sample size was 95 lesions and 17 lesions separately, and the pooled R0 resection rate was 83.1% (95% CI 76.3–89.9, I^2^ = 0%) and 48.6% (32.0–65.1, I^2^ = 0%), respectively.

## Validation of meta-analysis results

### Sensitivity analysis

We removed one research at a time and examined its impact on the primary outcomes estimate to see whether any one study had a dominant effect on the meta-analysis, and no single study had a substantial impact on the results.

### Heterogeneity

The dispersion of the computed rates was evaluated using the I^2^ percentage values, and the causes of heterogeneity were discovered using subgroup analysis. Overall, No heterogeneity was noted in delayed-bleeding events, and recurrence was in low heterogeneity. Intraoperative bleeding, en-bloc, and R0 resection rates were considered to be in substantial heterogeneity. Polyps size was the source of heterogeneity in the R0 resection rate by subgroup analysis.

### Publication bias

Publication bias assessment was carried out only in R0 resection rate, for the metaprop program is unable to do so. There was no indication of publication bias according to quantitative analysis utilizing the Egger regression test.

## Discussion

The objective of this systematic review was to assess the effectiveness and safety of underwater endoscopic mucosal resection (UEMR) for SNADETs ≤20 mm, which has not been performed before. Our analysis showed that the en-bloc resection rate of UEMR was 88.2% and the R0 resection rate was 69.1%. The pooled intraoperative bleeding rates and the delayed-bleeding rates were 2.9 and 0.9%, respectively, with only one patient happened delayed perforation and no intraoperative perforation. And the recurrence rate was 1.5%. Results indicated it was effective and safe to remove ≤20 mm SNADETs by utilizing the effect of floating force to fill the duodenal lumen with water.

Although conventional EMR is the most frequently used to treat ≤20 mm SNADA, it can be challenging due to a bending lumen in the duodenum, limited scope mobility, and the danger to inject submucosal for the muscle layer is extremely thin. Previous studies showed an R0 resection rate of 72–95.2% for EMR in small SNADETs (21, 27) compared with UEMR, but its high adverse events rate of 6.4% limited its security (10). As a new technology, UEMR can overcome these difficulties by immersion water into the intestinal, producing a natural optical magnifying effect that enhances the ability to see fine structural and microvascular details as well as the lesion’s delineation margin and enabling to improve the rate of en-bloc resection and R0 resection (30). Since polyps “float” away from the muscularis propria when filled with water without submucosal injection, this potentially decreases perforation and bleeding. For the recurrence, the guideline indicated that the risk of recurrence was related to the R0 resection which means a pathologically negative polyp border (31). In our analysis, a high R0 resection rate of 69.1% and a low recurrence rate of 1.5% were reported.

A meta-analysis of 8 studies with 258 polyps showed an 84.6% rate of en-bloc resection and a 6.9% rate of adverse events using UEMR for SNADETs (32). However, this study did not evaluate the R0 resection rate. The size of the polyps could influence the success of resection, especially when larger than 20mm, in multiple studies. Kato et al. (10) explored the en-bloc resection rate was only 30% in >20 mm SNADETs and ranged from 62.0 to 93.3% in ≤20 mm SNADETs. By comparison, all the lesions in our analysis were ≤20 mm, and we summarized all clinical outcomes of UEMR.

In the subgroup analysis according to the sample size, we found that the R0 resection rate and en-bloc resection rate were higher in the small samples subgroup (R0 resection rate 74.5 vs. 67.0%; en bloc resection rate 95.9 vs. 83.6%). The possible reason was the sampling error, small sample studies mean that they may not be representative of the whole, which makes the results calculated based on the sample unreliable. In the subgroup analysis according to tumor size, there were significant differences between the ≤10 and 10–20 mm SNADETs subgroups (R0 resection rate 83.1 vs. 48.6%; en bloc resection rate 100.0 vs. 84.0%). Thus, we surmised that tumor size was a risk factor for both R0 and en-bloc resection. This theory was supported by Li et al. (33) study, and indicated that polyp size was an independent factor determining resection outcome. In addition, since the I^2^ in subgroups were both 0%, lesion size was considered to be the source of heterogeneity in the R0 resection rate. Nevertheless, the cause of heterogeneity in the en-bloc resection rate was not clear.

There are several strengths to our review. Firstly, we comprised comprehensive literature searches containing ClinicalTrials.gov. with clear inclusion criteria, included studies with meticulous data extraction, rigorous assessment of study quality, and used different statistical methods according to the characteristics of the data. Secondly, according to the NOS quality evaluation system, all of the literature we included was of medium to high quality. Thirdly, we thoroughly evaluated the effectiveness and safety of UEMR for ≤20 mm polyps in non-ampullary duodenal and made subgroup analyses according to sample size and polyp size. Therefore, our findings might be more useful for clinical practice. Fourthly, heterogeneity in en-bloc resection and R0 resection was reduced by stratifying according to the size of tumors. In addition, for R0 resection, no publication bias was found and the outcome was confirmed to be stable and reliable by sensitivity analysis.

This study also has some limitations, most of which are inherent to any meta-analysis. First, all of the studies we included were cohort studies from Japan, thus, the findings of this study could not properly represent the effectiveness and safety of UEMR global. Second, even though we had conducted a comprehensive search of the literature, only 10 studies were included and four studies had a sample size of less than 50, both of which resulted in our final sample size not being sufficient. Third, because of some extremes in the data, we must use metaprop software, and the sensitivity analysis and publication bias analysis were not available. Fourth, subgroup analysis for polyp size was performed with a cut-off of 10 mm only, without more detailed stratification. To more accurately evaluate the efficacy and safety of UEMR in the resection of SNADETs, we propose that researchers further stratify the size of ≤20 mm polyps in the future. Fifth, because there have only been a few small sample clinical studies comparing UEMR, CEMR, and ESD, we decided to simply analyze UEMR’s efficacy for the removal of SNADETs in this study and not compare it to the other endoscopic resection methods. Sixth, different histological polyps may influence the efficacy and safety of the endoscopic resection (34), however, due to the lack of corresponding data in the included literature, we did not do the subgroup analysis.

Underwater endoscopic mucosal resection is an effective technique for SNADETs, and the rates of adverse events and recurrences are acceptable. Thus, the results of the meta-analysis suggest that UEMR should be recommended for optional treatment for SNADETs measuring ≤20 mm.

## Data availability statement

The original contributions presented in this study are included in the article/Supplementary material, further inquiries can be directed to the corresponding author.

## Author contributions

JL and SD contributed to the literature search and wrote the manuscript. JL, HP, YW, and YK participated in the statistic analysis or interpretation. JL and SY reviewed and edited the manuscript. SY was the guarantor of the work. JL and SD were the major contributors to finishing the manuscript. All authors read and approved the final manuscript.

## Supplementary material

Supplementary Figure 1 Forest plot, Intraoperative bleeding rate.

Supplementary Figure 2 Forest plot, delayed-bleeding rate.

Supplementary Figure 3 Forest plot, recurrence rate.

Supplementary Figure 4 Forest plot, en-bloc resection rate, subgroup analysis according to sample size.

Supplementary Figure 5 Forest plot, R0 resection rate, subgroup analysis according to sample size.

## Appendix 1

Literature search strategy:

Strategy:

PubMed: (((((Endoscopic Mucosal Resection[MeSH Terms]) OR (underwater endoscopic mucosal resection[Title/Abstract])) OR (UEMR[Title/Abstract])) OR (Underwater EMR[Title/Abstract])) OR (UW-EMR[Title/Abstract])) AND (((Duodenum[MeSH Terms]) OR (Duodenums[Title/Abstract])) OR (Duodenal[Title/Abstract])).

EMBASE: (“endoscopic mucosal resection”/exp OR “underwater endoscopic mucosal resection”: ab,ti OR uemr: ab,ti OR “underwater emr”: ab,ti OR “uw emr”: ab,ti) AND (duodenums: ab,ti OR duodenal:ab,ti OR “duodenum”/exp).

SCOPUS: ((TITLE-ABS-KEY (duodenum) OR TITLE-ABS-KEY (duodenums) OR TITLE-ABS-KEY (duodenal))) AND ((TITLE-ABS-KEY (endoscopic AND mucosal AND resection) OR TITLE-ABS-KEY (underwater AND endoscopic AND mucosal AND resection) OR TITLE-ABS-KEY (uemr) OR TITLE-ABS-KEY (underwater AND emr) OR TITLE-ABS-KEY (uw-emr))).

Clinical trials: (Endoscopic Mucosal Resection OR underwater endoscopic mucosal resection OR UEMR OR Underwater EMR OR UW-EMR) AND (Duodenum OR Duodenums OR Duodenal).

## Appendix 2

**APPENDIX TABLE 1:** MOOSE checklist for meta-analyses of observational studies.

Item no	Recommendation	Reported on Page no
**Reporting of background should include**		
1	Problem definition	3
2	Hypothesis statement	–
3	Description of study outcome(s)	4
4	Type of exposure or intervention used	3
5	Type of study designs used	3
6	Study population	3
**Reporting of search strategy should include**		
7	Qualifications of searchers (e.g., librarians and investigators)	3, Appendix 3
8	Search strategy, including time period included in the synthesis and key words	3, Appendix 3
9	Effort to include all available studies, including contact with authors	3
10	Databases and registries searched	3
11	Search software used, name and version, including special features used (e.g., explosion)	3–4
12	Use of hand searching (e.g., reference lists of obtained articles)	–
13	List of citations located and those excluded, including justification	–
14	Method of addressing articles published in languages other than English	3
15	Method of handling abstracts and unpublished studies	3
16	Description of any contact with authors	–
**Reporting of methods should include**		
17	Description of relevance or appropriateness of studies assembled for assessing the hypothesis to be tested	3–4
18	Rationale for the selection and coding of data (e.g., sound clinical principles or convenience)	4
19	Documentation of how data were classified and coded (e.g., multiple raters, blinding and interrater reliability)	4
20	Assessment of confounding (e.g., comparability of cases and controls in studies where appropriate)	4, 6
21	Assessment of study quality, including blinding of quality assessors, stratification or regression on possible predictors of study results	4, 6, 7
22	Assessment of heterogeneity	7
23	Description of statistical methods (e.g., complete description of fixed or random effects models, justification of whether the chosen models account for predictors of study results, dose-response models, or cumulative meta-analysis) in sufficient detail to be replicated	3
24	Provision of appropriate tables and graphics	Table 1, Supplementary materials
**Reporting of results should include**		
25	Graphic summarizing individual study estimates and overall estimate	Supplementary materials
26	Table giving descriptive information for each study included	Table 1
27	Results of sensitivity testing (e.g., subgroup analysis)	6–7
28	Indication of statistical uncertainty of findings	7
